# Tumor Connectomics: Mapping the Intra-Tumoral Complex Interaction Network Using Machine Learning

**DOI:** 10.3390/cancers14061481

**Published:** 2022-03-14

**Authors:** Vishwa S. Parekh, Jay J. Pillai, Katarzyna J. Macura, Peter S. LaViolette, Michael A. Jacobs

**Affiliations:** 1 The Russell H. Morgan Department of Radiology and Radiological Science, School of Medicine, The Johns Hopkins University, Baltimore, MD 21205, USA; vishwaparekh@jhu.edu (V.S.P.); jpillai1@jhmi.edu (J.J.P.); kmacura@jhmi.edu (K.J.M.); 2 Department of Computer Science, The Johns Hopkins University, Baltimore, MD 21210, USA; 3 Department of Neurosurgery, School of Medicine, The Johns Hopkins University, Baltimore, MD 21287, USA; 4 Sidney Kimmel Comprehensive Cancer Center, School of Medicine, The Johns Hopkins University, Baltimore, MD 21205, USA; 5 Department of Radiology, Medical College of Wisconsin, Milwaukee, WI 53226, USA; plaviole@mcw.edu

**Keywords:** tumor connectomics, multiparametric MRI, graph theory, complex networks, cancer, breast, brain, prostate

## Abstract

**Simple Summary:**

Understanding the complex network of high-level relationships within tumors and between surrounding tissue is challenging and not fully understood. Our findings demonstrate that the tumor connectomics framework (TCF) models different networks within the tumors and surrounding tissue that are detectable on imaging. The TCF extracts a set of graph network features for each lesion and provides insight into the different types of interactions of a cancer under investigation. These TCF networks are visualized with the radiological parameters and overlaid onto the structural images for better understanding of the global and regional connections within the lesion and surrounding tissue. This information could be used for improved cancer therapeutic targeting in neoplasms and response within different organ systems.

**Abstract:**

The high-level relationships that form complex networks within tumors and between surrounding tissue is challenging and not fully understood. To better understand these tumoral networks, we developed a tumor connectomics framework (TCF) based on graph theory with machine learning to model the complex interactions within and around the tumor microenvironment that are detectable on imaging. The TCF characterization model was tested with independent datasets of breast, brain, and prostate lesions with corresponding validation datasets in breast and brain cancer. The TCF network connections were modeled using graph metrics of centrality, average path length (APL), and clustering from multiparametric MRI with IsoSVM. The Matthews Correlation Coefficient (MCC), Area Under the Curve-ROC, and Precision-Recall (AUC-ROC and AUC-PR) were used for statistical analysis. The TCF classified the breast and brain tumor cohorts with an IsoSVM AUC-PR and MCC of 0.86, 0.63 and 0.85, 0.65, respectively. The TCF benign breast lesions had a significantly higher clustering coefficient and degree centrality than malignant TCFs. Grade 2 brain tumors demonstrated higher connectivity compared to Grade 4 tumors with increased degree centrality and clustering coefficients. Gleason 7 prostate lesions had increased betweenness centrality and APL compared to Gleason 6 lesions with AUC-PR and MCC ranging from 0.90 to 0.99 and 0.73 to 0.87, respectively. These TCF findings were similar in the validation breast and brain datasets. In conclusion, we present a new method for tumor characterization and visualization that results in a better understanding of the global and regional connections within the lesion and surrounding tissue.

## 1. Introduction

Complex graph network analysis is the study of intricate, irregular, and dynamic networks that are evolving in time and graph network research has received considerable attention since the seminal paper by Erdos and Renyi on random graphs [1]. The studies by Watts and Strogatz on small world phenomenon and by Barabasi and Albert on scale free property have sparked an increased interest in the field of dynamically evolving complex network analysis [2,3,4]. Complex network analysis has shown incredible success in different areas in the analysis of social, world-wide web, brain, gene, and other networks [3,5,6,7,8,9,10,11,12,13]. We extend the use of complex network analysis for characterization of magnetic resonance imaging (MRI) of normal and lesion tissue in different types cancers. Other studies using MRI in these types of relationships are in brain lesions using both task-based functional MRI (fMRI) and resting state fMRI.

Functional brain networks have been studied using both task-based MRI and resting state fMRI. The applications of fMRI in brain cancer have looked at local disruptions or alterations of brain networks by lesions or pathological disorders as important aspects of presurgical brain mapping and therapeutic planning. However, identification of the different brain networks and their disruption of function requires supervision or parcellation of the different brain regions [12,14,15,16,17,18].

**Figure 1:**
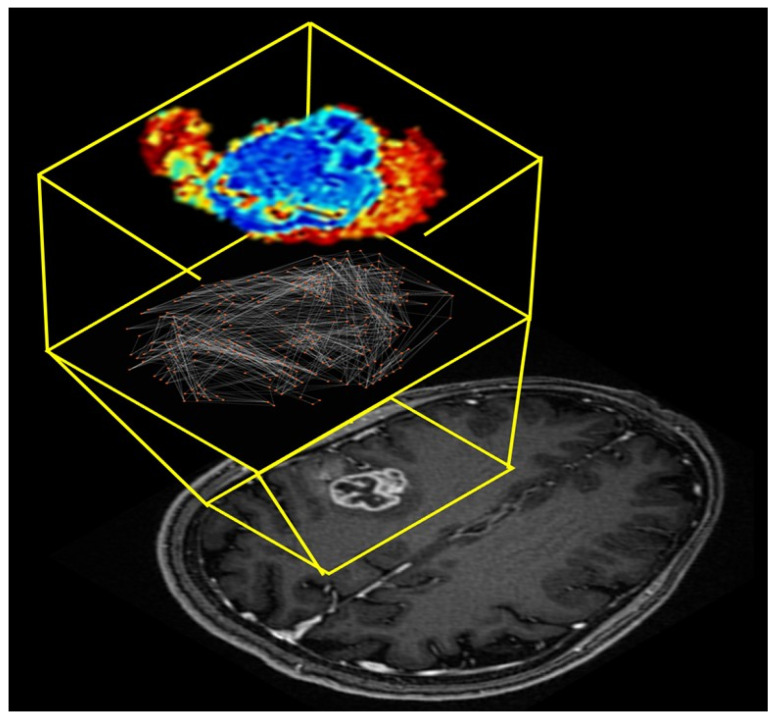
Illustration of the tumor connectome framework overlaid onto an axial T_1_-weighted post contrast MRI. The tumor connectome framework was modeled from the multiparametric brain MRI dataset of a patient with a right side perirolandic WHO Grade 4 glioblastoma with IDH1 wild type and MGMT- unmethylated. The magnified yellow insert demonstrates the lesion (bottom), the intra- ana extra-tumoral connections (middle) and visualization of the complex network metrics.

One of the current state-of-the-art methods for extraction of intra-tumoral heterogeneity from radiological images are based on radiomic texture analysis, which provides quantitative information about the intensity distribution and inter-voxel relationships within regions of interest drawn on radiological images [19,20,21,22,23] or full images [24,25]. These radiomic methods, however, do not uncover the underlying network structure of the tumor or the relationship with the lesion and surrounding tissue. In addition, visualization and interpretation of traditional texture analysis methods can be difficult and still under investigation [26]. Tumors are extremely heterogeneous and consist of many tumor microenvironments or clusters. These tumor microenvironments have sometimes been recently referred to as habitats and investigated in multiple studies [27,28,29,30,31,32,33,34,35]. However, the habitats are obtained by supervised segmentation of the tumor into a fixed set of groups, which may not reflect the true organizational structure of the tumor. Therefore, representing the tumor as a network structure will provide increased interpretability, quantification, and visualization of the different tumor substructures. By modeling the tumor as a complex network would allow for unbiased evaluation of the tumor microenvironment without assumption of a fixed classes or hard segmentation boundaries. This new approach could provide important insight into the biological organizational structure of the tumor network. Furthermore, extending the evaluation of how the complex network structure evolves over time with or without treatment could provide new metrics for planning or evaluation of treatment response in patients. In this work, we develop and extend the application of complex network analysis to the intra-tumoral network formed from integration of multiparametric (mp) radiological imaging of cancer [13]. We establish a tumor connectomics framework (TCF) for complex network analysis of tumors and surrounding tissue using mpMRI and demonstrate that the TCF performance in classification of breast, brain, and prostate lesions. Figure 1 demonstrates a typical tumor connectome from mpMRI of the brain in a patient with a WHO Grade 4 glioblastoma (malignant primary brain tumor). The anatomic T1-weighted post contrast MRI image is shown for visualization of the lesion and the intra-tumoral connections with surrounding tissue.

## 2. Materials and Methods

### 2.1. Tumor Connectomics Framework

The tumor connectomics framework (TCF) models either a single or multidimensional imaging dataset into a complex network model. In the TCF model, the nodes in the graph are represented by different voxels within each image and the edges correspond to the relationship between these voxels and image structure. The edge weights are determined by a user defined similarity metric, such as correlation coefficient or geodesic distance between the voxels in each image. Mathematically, let radiological imaging dataset can be represented as the set. $\;X_{tissue\;signature}=\left\{x_{11},x_{12},\;\ldots ,\;x_{mn}\right\}\subset R^{D}$ where, *x_mn_* is the tissue signature at voxel position (i,j), *m* and *n* are the number of rows and columns in each image within the imaging dataset and *D* is the image space defined by number of imaging parameters or modalities used in the TCF. The TCF analyzes the imaging dataset, *X*, using the following procedure:

1. In the first step, a pairwise Euclidean distance matrix is computed for the D dimensional image space.

2. The second step involves identifying the nearest neighborhood of every point in the image space, X. This is carried out by identifying the k nearest neighbors or setting a predefined threshold, t, for closeness of each neighboring point.

3. The third step involves transforming the Euclidean distance matrix into a geodesic distance matrix and forming an adjacency matrix using the nearest neighborhood information extracted from step 2. The geodesic distance matrix is then used for visualizing and analyzing the intratumor or tumor connectome network.

#### 2.1.1. Complex Network Analysis

4. The geodesic distance matrix is analyzed using graph theoretic centrality metrics such as degree centrality, eigenvector centrality, and betweenness centrality to identify the different hubs within the tumor. Next, cluster analysis is applied using a clustering coefficient and connected component analysis to evaluate the different subgraphs within the tumor connectome. Then, global metrics of average path length, average degree centrality, and connectivity are computed to evaluate the overall structure of the underlying complex network.

#### 2.1.2. Visualization

5. The TCF connections obtained from step 3 are overlaid onto a radiological image for visualization of tumor microenvironments and functional connections creating the TCF map. The edges on the TCF map represent the edges obtained after implementing the nearest neighborhood cut-off determined either by k-nearest neighbors or threshold, t.

To calculate the geodesic distances, the images are normalized to the range of zero to one using double precision to minimize the loss of information. The graph centrality metrics were modified from their traditional definition and normalized to account for the variability in tumor sizes [7]. However, the average path length metric was only computed as a local metric overlaid on the tumor. The tumor connectome is not always fully connected and may have more than one distinct subgraph (or multiple clusters). For such a graph, the value of average path length would be infinite. To that end, the number of clusters was defined as an additional global metric for each tumor connectome.

#### 2.1.3. Multivariate Patient Classification

6. The multivariate patient classifier was modeled using the combined IsoSVM algorithm. The IsoSVM algorithm is a hybrid machine learning algorithm for classification of datasets that could not be separated by a linear hyperplane. The Isomap algorithm acts as the nonlinear kernel by first transforming the high-dimensional non-linear data into a low-dimensional linearly separable space by preserving the local neighborhood structure. In the next step, the SVM model is trained on the transformed data for the purpose of classification. The graph theoretic metrics of degree centrality, betweenness centrality, eigenvector centrality, average path length, node strength, and clustering coefficient form the input high-dimensional classification dataset extracted from any input tumor imaging data. The IsoSVM algorithm is implemented on the graph metrics dataset to classify different tumor connectomes into different classes as defined by the underlying application. The training data may be imbalanced in one or the other class. This imbalance in the classes can cause the trained model to be biased towards the class with a higher number of training samples. The class imbalance between the two groups in each of the patient cohort was resolved by using separate misclassification penalties for the two groups. All the IsoSVM hyperparameters were optimized using grid-search and evaluated using leave-one-out cross validation.

### 2.2. Clinical Data

#### 2.2.1. Breast mpMRI Dataset

In this case, 100 (59 with malignant and 41 with benign lesions) were included in this retrospective study. Breast lesions were categorized by cancer type and histological phenotyping based upon hormonal markers by tissue samples obtained at biopsy. Estrogen and progesterone receptors (ER and PR), HER2-Nu by FISH, and Ki-67 proliferation index (%) were carried out on each patient.

Multiparametric Breast sequences: Patients were scanned on a 3T MRI system (3T Achieva, Philips Medical Systems, Best, The Netherlands) using a bilateral, dedicated phased array breast coil (InVivo, Orlando, FL, USA) with the patient in the prone position. Total imaging time was approximately 45 min.

Proton MRI Imaging. T_2_-weighted spin echo (TR/TE/IR = 7142/70/220 ms, Field of View (FOV) = 350 × 350, matrix = 220 × 195, slice thickness (ST) = 5 mm, SENSE = 2 and Averages (Ave) = 2) and fast spoiled gradient echo (FSPGR) T_1_-weighted (TR/TE = 5.4/2.3 ms, FOV = 350 × 350, matrix = 548 × 550, ST = 3 mm, SENSE = 2 and Ave = 1) sequences were acquired.

Pharmacokinetic Dynamic Contrast-Enhanced MRI. The Pharmacokinetic (PK) DCE was obtained using non-fat-suppressed (FS), three-dimensional (3D), FSPGR T_1_-weighted (TR/TE = 3.4/1.7 ms, FOV = 350 × 350, matrix = 256 × 126, Flip angle = 10, slice thickness = 5 mm, and Ave = 1) sequences. Images were acquired pre- and 14 post- intravenous administration of a GdDTPA contrast agent (0.2 mL/kg (0.1 mmol/kg)) via a power injector at a rate of 2 mL/s, with temporal resolution of 15 sec. Post-processing of the DCE exam was performed by a combined Brix and Tofts model [36] using DynaCad (InVivo) software from the identified breast lesions.

High Resolution Dynamic Contrast-Enhanced MRI. T_1_-weighted 3D GRE with FS (TR/TE = 5.8/2.9 ms, FOV = 350 × 350, matrix = 720 × 720, Flip angle = 13, ST = 3 mm, and Ave = 1) were obtained pre and post the PK-DCE.

Diffusion weighted Imaging. Diffusion-weighted imaging was acquired before contrast imaging using an FS fast spin echo planar parallel imaging sequence (TR/TE/IR = 9548/70 ms, FOV = 350 × 350, matrix = 220 × 195, SENSE = 2, Ave = 2, ST = 3 mm, b = 0, 200, 600, 800 s/mm^2^) on three planes and in less than three minutes. Trace apparent diffusion coefficient (ADC) maps were constructed using a diffusion monoexponential model [37].

#### 2.2.2. Brain mpMRI Dataset

In this case, 24 patients with 24 brain tumors were imaged in this retrospective study. Of the 24 patients, nine patients had WHO Grade 2 brain tumors and 15 patients had WHO Grade 4 brain tumors. Histopathological samples were acquired during surgery. The tumor grade was determined and genomic profile markers were recorded when available. The molecular markers, when performed, were isocitrate dehydrogenase gene (IDH1) mutation status, ATP-dependent helicase (ATRX), p53, 1p/19q co-deletion, O6-methylguanine-DNA methyltransferase (MGMT) methylation status.

Brain mpMRI Sequences: MR images were obtained using a 3.0 Tesla Siemens Trio Tim system (Siemens Medical Solutions, Erlangen, Germany) with a 12-channel head matrix coil. Multiparametric MRI included a 3D T_1_-MPRAGE sequence (TR = 2300 ms, TI = 900 ms, TE = 3.5 ms, flip angle = 9°, field of view(FOV) = 24 cm, acquisition matrix = 256 × 256 × 76, ST = 1 mm), a 2D T_2_-FLAIR axial sequence (TR = 9310 ms, TI = 2500 ms, TE = 116 ms, FOV= 24 cm, acquisition matrix = 320 × 240 × 50, ST = 3 mm). Diffusion weighted imaging was obtained using spin echo EPI parallel imaging sequence TR/TE = 6700/90, flip angle = 90°, 192 × 192 mm FOV, Matrix-96 × 96, ST = 3 mm, b values = 0 and 1000 s/mm^2^. Trace monoexponentially Apparent Diffusion Coefficient (ADC) of water maps were constructed from the DWI. ADC map values were obtained from the central tumor and peri-tumoral or peripheral regions.

#### 2.2.3. Prostate mpMRI Dataset

In this case, 15 patients with prostate cancer were evaluated retrospectively. The TCF was tested on a cohort of male patients with either Gleason 6 (3 + 3) or Gleason 7 (3 + 4) prostate tumors [38]. Patients were enrolled in an ongoing study at the Medical College of Wisconsin, where additional research imaging was acquired, in addition, to their clinical standard of care imaging prior to prostatectomy. Post-surgery, the prostate tissue was sectioned in the same orientation as the MRI was acquired using patient specific 3D-printed slicing jigs [39,40]. Samples were then whole mount stained and annotated using the Gleason pattern criteria [38].

Prostate MRI Sequences: The MRI was acquired on a 3 T scanner (General Electric, Waukesha, WI, USA) using an endorectal coil. The mpMRI included field of view (FOV)–optimized and Constrained Undistorted Single shot (FOCUS) DWI, DCE imaging, and T_2_-weighted imaging. T_2_ acquisition parameters were as follows: repetition time (TR/TE) = 4360/125 ms, FOV = 120, Matrix-512 × 512, ST = 3 mm. Diffusion images (TR/TE = 4000/69 ms, Matrix-256 × 256, ST = 4 mm) were collected with ten b-values (b = 0, 10, 25, 50, 80, 100, 200, 500, 1000, 2000). Monoexponential ADC maps were created [41].

#### 2.2.4. Independent Validation Datasets

##### Breast MRI-Neoadjuvant Chemotherapy Data Set

The first independent dataset was the Breast-MRI-NACT-Pilot dataset for treatment response prediction of breast cancer obtained from the University of California San Francisco (UCSF) ISPY study [42,43]. The validation dataset consisted of 34 patients who underwent neoadjuvant chemotherapy, with multiparametric MRI acquired at both baseline and after first treatment cycle. Treatment response was defined by clinical standards and used for ground truth. The clinical response metric (CR) is defined in four categories: (CR = 1), no evidence of disease, 2. (CR = 2), greater than 1/3 decrease in clinically longest diameter (LD) 3. (CR = 3) less than 1/3 decrease in LD and progressive disease (PD) (CR = 4). The MRI scans were obtained on a 1.5 T magnet in the sagittal orientation using a dedicated breast RF coil. The MR images obtained were fat suppressed, T_1_-weighted dynamic contrast enhanced series obtained unilaterally in the sagittal orientation with TR ≤ 20 ms, TE = 4.5 ms, Flip Angle ≤ 45°, FOV: 16–18 cm, matrix size > 256 × 192, ST ≤ 2.5 mm. The tumor connectomics framework was applied at the baseline study and after the first cycle (D7) of NACT. The percentage difference between the measurements at the two timepoints, delta-TCF was computed and correlated to clinical response that were categorized into two groups (CR = 1, 2 vs. CR = 3, 4).

##### Brain mpMRI Dataset

The brain mpMRI independent validation dataset consisted of 443 subjects with low- and high-grade gliomas (LGG, HGG, respectively) acquired from two data repositories on the Cancer Imaging Archive (TCIA) [42,44,45,46,47]. The first dataset consisted of 159 patients with 105 WHO Grade 2 and 54 WHO Grade 4 gliomas. The dataset was originally acquired for prediction of 1p/19q co-deletion using multiparametric MRI [42,44,45,46,47]. There were 97 patients with oligoastrocytoma, 45 with oligodendrogliomas, and 17 patients with astrocytomas. Of the 159 subjects, 102 patients had biopsy proven non-deleted (*n*/*n*) and 57 patients had biopsy proven co-deleted (*d*/*d*) LGG, computed using FISH. The mpMRI consisted of 3-mm-thick and T_2_-weighted images and 1-mm-thick axial T_1_ pre and post contrast spoiled-gradient recalled images, acquired at 1.5T or 3T. The complete acquisition details can be found in reference [46].

The second dataset comprised of 285 patients from the brain tumor segmentation (BRATS 2017) challenge dataset. Of the 285 patients, 210 patients were designated HGG and 75 patients were designated LGG. No pathological or molecular markers were provided. The mpMRI consisted of pre and post 3D T_1_-weighted, 2D T_2_-weighted, and T_2_-weighted FLAIR images, complete MRI acquisition parameters can be found in references [44,45]. The tumor connectomics framework was applied to the tumor boundaries supplied in the dataset used for segmentation of the axial slice with the largest tumor diameter of each patient across the two datasets for classifying LGG from HGG.

### 2.3. Statistical Analysis

Summary statistics (mean and standard deviation of the mean) were computed for each of the graph metrics. A two-sided t-test was performed to compare the different tumor groups (benign vs. malignant breast, Grade 2 vs. Grade 4 brain tumors, etc.). We used the Matthews Coefficient of Correlation (MCC) for comparison between each of the groups [48,49]. We computed the area under the receiver operating characteristic (ROC) curve (AUC-ROC) and the area under the precision-recall (PR) curve (AUC-PR) for each of the graph metrics [50,51]. Statistical significance was set at *p* < 0.05.

## 3. Results

### 3.1. Breast Tumor Connectomics

**Figure 2:**
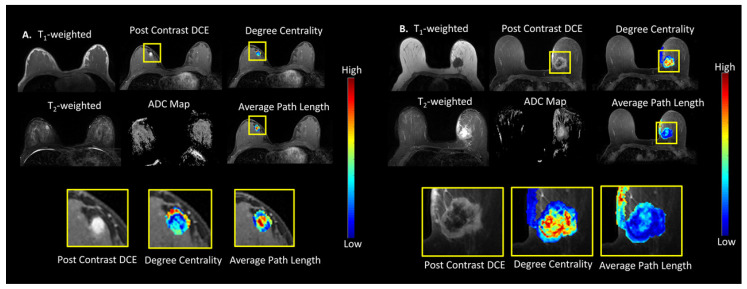
Tumor connectomics of degree centrality and average path length maps overlaid on multiparametric breast MRI. (**A**) An Example of a benign lesion in a 27-year-old female with focal fibroadenomatous change. The Apparent Diffusion Coefficient (ADC) Map value for this lesion was 1.54 × 10^−3^ mm^2^/s. (**B**) Example of an infiltrating poorly differentiated ductal carcinoma in a 56-year-old female within left breast. The ADC Map value for the lesion was 0.68 × 10^−3^ mm^2^/s.

**Figure 3:**
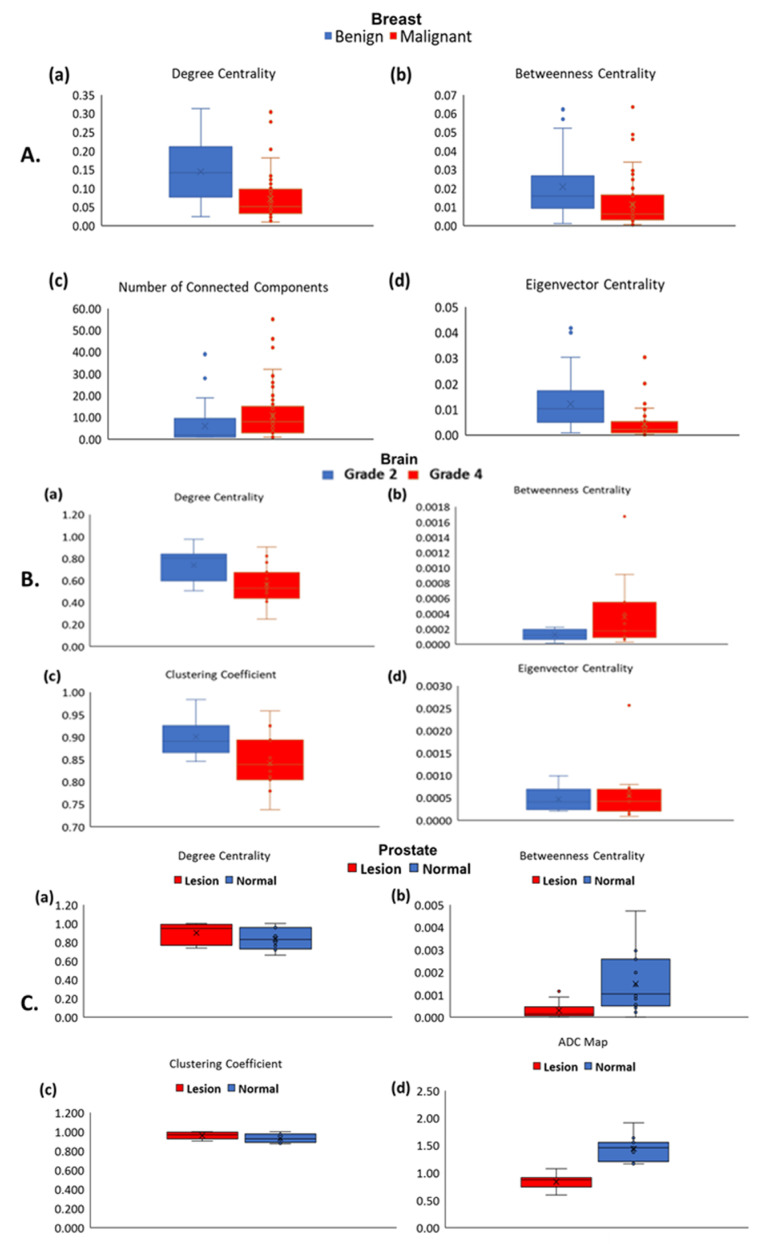
Box Plots of the tumor connectomics metrics with mean with standard error of the mean are shown in the different cancers. (**A**) Breast tumor connectomics are shown for benign (blue) and malignant lesions (red) for the different graph centrality and the connectivity metrics computed from the breast mpMRI of (**a**) degree centrality, (**b**) betweenness centrality, (**c**) clustering coefficient, and (**d**) eigenvector centrality. (**B**) Brain tumor connectomics are shown for WHO Grade 2 (blue) and Grade 4 (red) lesions for the different graph metrics computed from the tumor connectome consisting of (**a**) degree centrality, (**b**) betweenness centrality, (**c**) clustering coefficient, and (**d**) eigenvector centrality obtained from tumor connectome. (**C**) Prostate lesion tumor connectomics are shown normal (blue) and lesion (red) tissue within the prostate based on (**a**) degree centrality, (**b**) betweenness centrality, (**c**) clustering coefficient, and (**d**) apparent diffusion coefficient (ADC) map value obtained from tumor connectome.

The TCF was tested on a breast cancer patient cohort of 100 patients. The mean age of the patients was 52 ± 11 years old (range = 22–80) and the histopathology lesion tumor type and hormonal molecular markers are summarized in Table 1 and Table 2 details the graph metrics of the benign and malignant lesions. Figure 2A,B show the TCF maps for degree centrality and average path length from representative patients with benign and malignant lesions. The box plots of the TCF metrics are shown in Figure 3A. There were significant differences (*p* < 0.001) between the ADC map values for malignant (1.16 ± 0.25 × 10^−3^ mm^2^/s) and benign lesions (1.64 ± 0.38 × 10^−3^ mm^2^/s). Similarly, there were significant differences in areas with higher PK-DCE values of K^trans^ for malignant lesions (0.46 ± 0.35 (1/min)) than benign lesions (0.23 ± 0.18 (1/min)). There was increased average path length within the malignant lesions (3.28 ± 0.91) compared to benign lesions (2.73 ± 0.93). Each of the centrality metrics accurately classified benign lesions from malignant lesions with degree centrality (Table 2). However, the average path length was not significantly different within these lesions (*p* > 0.05). The benign lesions demonstrated significantly higher connectivity characterized by a high degree centrality (0.14 ± 0.08) and betweenness centrality (0.02 ± 0.02) compared to the malignant lesions with lower centrality metrics (0.07 ± 0.06, 0.01 ± 0.01). Each of the centrality metrics accurately classified benign lesions from malignant tumors with the degree centrality metric exhibiting the maximum AUC-ROC (area under the receiver operating characteristic curve) of 0.79 (95% CI = 0.70–0.89) with AUC-PR (area under the precision-recall curve) of 0.83 (95% CI = 0.72– 0.90) with a strong Matthews Correlation Coefficient (MCC) of 0.54. The IsoSVM algorithm achieved sensitivity and specificity of 76% and 88%, respectively, with an AUC-ROC of 0.86 (CI = 0.76–0.92) and AUC-PR of 0.90 (95% CI = 0.79–0.95) with a strong MCC of 0.63.

### 3.2. Brain Tumor Connectomics

**Figure 4:**
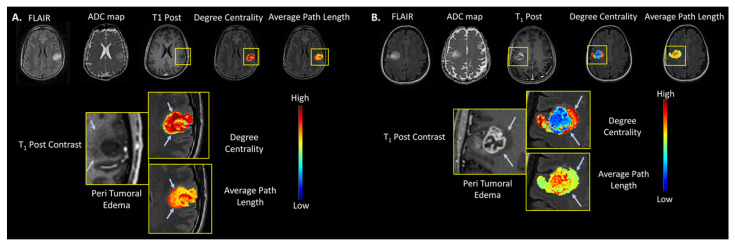
Examples of a multiparametric MRI in WHO Grade 2 and 4 brain lesions. (**A**) Example of a WHO Grade 2, IDH1 mutant and 1p/19q-codeleted, left perirolandic oligodendroglioma and the corresponding degree centrality and average path length maps, overlaid on T1-post contrast anatomic images. The TCF corresponding to the peripheral region of this FLAIR-hyperintense infiltrative tumor that demonstrates less prominent T_1_ hypointensity than the central core portion of the tumor is shown by the light blue arrows. Notice the more homogeneous appearance of the TCF in this low-grade tumor compared to that of the high-grade tumor shown on the right. (**B**) Example of a WHO Grade 4 IDH1 wild type and MGMT unmethylated right perirolandic glioblastoma. The maps of tumor connectomics metrics degree centrality and average path length are overlaid on the T_1_-post contrast anatomic images displaying the tumor. The nonenhancing T_2_ FLAIR-hyperintense region, which reflects peritumoral edema and microscopic tumor infiltration, is shown by the light blue arrows.

The TCF was tested on a cohort of 24 patients with de novo brain tumors who were undergoing preoperative MRI, with mean age of 51 ± 15 years. There were 13 male and 11 female patients. Of the 24 patients, nine patients had a Grade 2 (37.5%) and 15 patients (62.5%) had a Grade 4 tumor. The WHO 2016 molecular markers were distributed as follows in these patients: IDH mutation was found in ten Grade 2 and two Grade 4 lesions, MGMT promoter methylation was present in four of the Grade 4 lesions and none found in Grade2. The 1p/19q co-deletion was detected in five Grade 2 and one Grade 4 lesion. The p53 was found in two Grade 2 and three Grade 4. The ATRX expression was detected in two Grade 2 and one Grade 4 lesions. For the brain tumor segmentation with FLAIR-defined edema, there were no significant differences (*p* = 0.22) between the ADC map values for Grade 4 (1.38 ± 0.34 × 10^−3^ mm^2^/s) and Grade 2 brain tumors (1.54 ± 0.29 × 10^−3^ mm^2^/s). In contrast, the ADC map values for brain tumor segmentations without peritumoral edema (i.e., central portions of the tumors) were significantly different (*p* = 0.002) between Grade 2 (1.58 ± 0.29 × 10^−3^ mm^2^/s) and Grade 4 (1.13 ± 0.25 × 10^−3^ mm^2^/s) brain tumors. Figure 4A,B show representative examples of the degree centrality and the average path length in WHO Grade 2 and Grade 4 tumors. In general, Grade 4 tumors demonstrated lower clustering coefficients and degree centrality compared to Grade 2 tumors. However, Grade 4 tumors demonstrated greater betweenness centrality and average path length than Grade 2 tumors. These graph metrics are summarized in Table 3 and box plots are shown in Figure 3B. The most predictive metric for classifying Grade 2 from Grade 4 brain tumors was the average path length with an AUC-ROC of 0.85 (95% CI = 0.69–1.00) and AUC-PR of 0.93 (95% CI = 0.82–0.98) and had a strong MCC (0.60). The corresponding sensitivity and specificity values were 60% and 100%, respectively.

### 3.3. Prostate Tumor Connectomics

**Figure 5:**
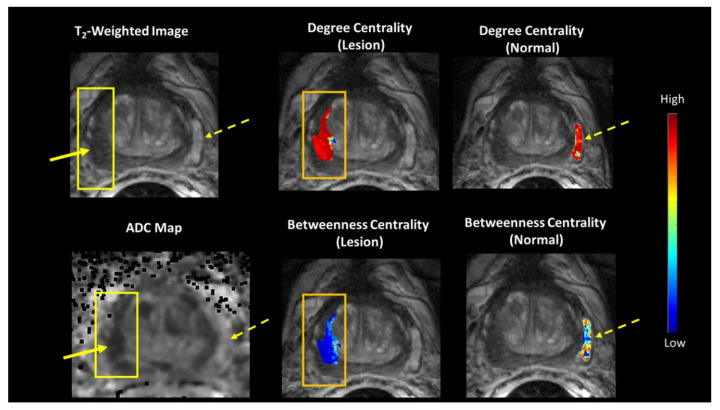
(**Left**) A representative patient with a Gleason 7 prostate lesion and corresponding mpMRI. (**Middle** and **Right**) The tumor connectomics framework (TCF) demonstrating the betweenness and degree centrality maps overlaid on normal (dotted yellow arrow) and lesion (solid yellow arrow) tissue corresponding with the T_2_-weighted image and Apparent Diffusion Coefficient (ADC) map.

The TCF was tested on a cohort of 15 patients with biopsy proven prostate tumor (Gleason 6 (3 + 3) or Gleason 7 (3 + 4), or Gleason 9 (5 + 4) and increased PSA (7.11 ± 4.49) who underwent mpMRI. These patients had an average age of 59 ± 6 years. Of the 15 patients, six patients (40%) had Gleason score 6 (G6), eight patients (53%) had Gleason score 7 (G7), and one patient (7%) had Gleason score 9. Figure 5 illustrates the betweenness and degree centrality maps from a prostate lesion and normal tissue of a representative patient overlaid on the T_2_-weighted and ADC map images. Normal prostate tissue exhibited higher betweenness and average path length compared to the prostate lesion tissue. Prostate lesions had a higher clustering coefficient. However, when G6 is compared to G7, G6 had significantly increased degree and eigenvector centrality and clustering coefficient. Whereas, G7 demonstrated significantly higher betweenness centrality and average path length and box plots are shown in Figure 3C. Table 4 and Table 5 summarizes these results of the different graph theoretic metrics between normal and prostate lesion tissue.

### 3.4. Independent Validation Datasets

#### 3.4.1. Breast MRI-Neoadjuvant Chemotherapy Data Set

**Figure 6:**
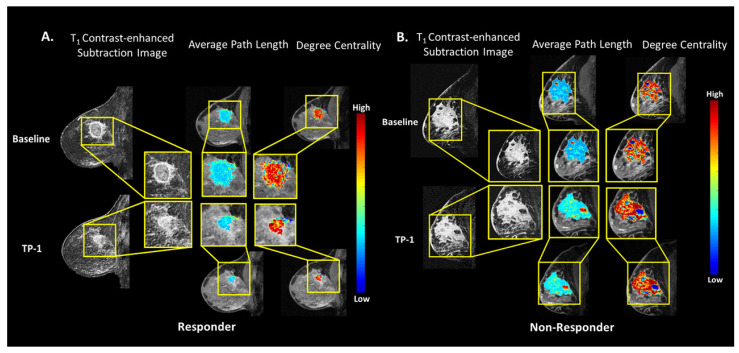
Examples of the breast MRI validation dataset in a responder and nonresponder patients after NACT. (**A**) Breast MRI in a typical responder patient that received NACT. The patient’s lesion decreased in size after NACT treatment. Shown are the overlays of the TCF metrics of degree centrality and average path length maps with decreases in the degree centrality and average path lengths. (**B**) Breast MRI in a nonresponder patient that received NACT with an increase in the patient’s lesion size after NACT. Shown are the overlays of the TCF metrics of degree centrality and average path length maps with increases in the degree centrality and average path lengths.

The TCF was tested on a cohort of 34 patients in the I-SPY 1 study with biopsy proven breast cancer that underwent neoadjuvant chemotherapy (NACT) with longitudinal monitoring using DCE MRI [43]. The clinical response was defined as CR1, no evidence of disease. CR2, greater than 1/3 decrease the clinical longest diameter (LD) of the lesion, CR3, less than 1/3 decrease in the LD, finally, CR4, stable disease or progressive disease. Of the 34 patients, eight patients (24%) had a CR1, 17 patients (50%) had CR2, seven patients (21%) had CR3, and two patients (0.06%) had CR4. The percent difference (delta TCF) between the TCF metrics were computed from mpMRIs obtained at baseline and after the first cycle of treatment for prediction of treatment response. Figure 6A,B show representative patients from each group with overlaid TCF metrics. Patients that had a response to NACT had a significant increase (*p* = 0.01) in the betweenness centrality (0.53 ± 0.50), compared to the patients who did not respond to NACT (−0.05 ± 0.50). The corresponding AUC-ROC of 0.84 (95% CI = 0.67–0.94) and an AUC-PR of 0.92 (95% CI = 0.73–0.98) and a very strong IsoSVM MCC of 0.70 was noted in the responders. Similar trends were observed in eigenvector centrality (EC) between responders and nonresponders ((EC_CR=1,2_ = 0.49 ± 0.38, EC_CR=3,4_ = 0.05 ± 0.56 *p* = 0.05, AUC-ROC = 0.76 (95% CI = 0.58–0.89), AUC-PR = 0.85 (95% CI = 0.66–0.95), Strong MCC = 0.50)). However, there was a decrease in the degree centrality between the responders and nonresponders with an AUC-ROC = 0.62 (95% CI = 0.44–0.78) and AUC-PR = 0.78 (95% CI = 0.58–0.90) with a weak MCC = 0.29. The TCF graph metrics are summarized in Table 6.

#### 3.4.2. Brain Dataset of Low vs. High Grade Gliomas

**Figure 7:**
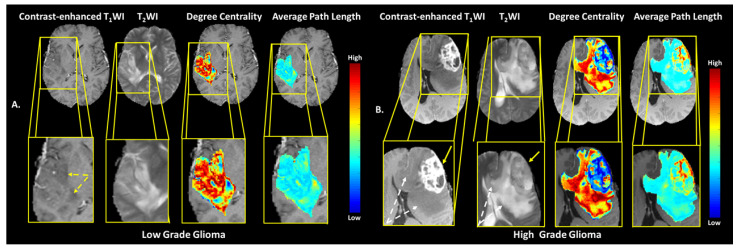
Examples of the brain MRI from the BRATS validation data set. (**A**) A right insular and superior temporal lobe low grade oligodendroglioma (yellow arrows) and the corresponding degree centrality and average path length maps overlaid on postcontrast T_1_ -weighted anatomic images. (**B**) A WHO Grade 4 left frontal lobe glioblastoma with the TCF maps of the metrics degree centrality and average path length overlaid on the postcontrast T_1_-weighted anatomic images displaying the tumor. The nonenhancing T_2_ -hyperintense peritumoral edema is shown by the dotted white arrows in the Grade 4 lesion.

The TCF was tested on an independent brain dataset consisting of 443 subjects with Low Grade Glioma (LGG) and High Grade Glioma HGG acquired from two data repositories on TCIA [43,45,46,48]. The TCF was applied to the tumor boundary segmented from axial slice with the largest tumor diameter from each patient’s lesion across the datasets for classifying HGG from LGG. There were significant results (*p* < 0.002) from the validation brain dataset compared to the original brain dataset with strong MCCs (0.40–0.46) and one weak MCC of 0.22. In general, HGG demonstrated a lower degree centrality compared to LGG (AUC-ROC and ACU-PR of 0.75 (CI = 0.70–0.79) and 0.82 (CI = 0.77–0.86) and strong MCC = 0.41) and the HGG demonstrated greater betweenness centrality and average path length than LGG ((AUC and ACU-PR of 0.61 (CI = 0.56–0.65) and 0.67 (CI = 0.61–0.72) and weak MCC = 0.22)). Finally, the IsoSVM results were similar with the initial brain dataset with an AUC-ROC and ACU-PR of 0.77 and 0.82, respectably and strong MCC of 0.46. The representative TCF of typical LLG and HGG tumors are shown in Figure 7A,B and the TCF graph metrics are summarized in Table 7.

## 4. Discussion

We have developed a tumor connectomics framework to explore the complex network connections within tumors and surrounding tissue for quantification, visualization, and analysis of the macro and micro tumor environment by using an advanced graph theoretic method. We demonstrated that the TCF potential for delineating different interactions between the two environments in several cancers using mpMRI. The TCF had excellent AUC-ROC, AUC-PR, and MCC values for breast lesion classification between benign and malignant lesions, distinguishing LGG from HGG brain tumors, and characterizing normal tissue and tumor regions within the prostate, both in test and validation datasets. There was distinct contrast between lesions and surrounding tissue for improved visualization of the intra-tumoral and peritumoral intricate network connections. These types of different connections could be used to identify and map specific characteristics within the subregions or microenvironment present within the lesions, while providing for quantification of different tissue types using graph metrics [4,7]. 

This TCF the tumor structure, vascularity distribution, and may be used for treatment planning mapping allows for the investigation of the multiple sub-regions within and around lesions that interact with each other within the TCF network model [52]. For example, malignant lesion TCF connectomes had significantly larger numbers of tumor microenvironments when compared to benign connectomes. Subgraph analysis of these tumor microenvironments could potentially provide further information regarding for different therapeutic interventions and for monitoring of response after therapeutic intervention. In particular, TCF mapping of brain tumor microenvironment may allow for radiation treatment planning with improved target detection based on the TCF overlays on the lesion areas. The spatial information may also prove to be useful in distinguishing true progression from pseudo progression following chemoradiation and/or immunotherapy in high grade brain tumors, which remains a major clinical challenge. Moreover, for brain lesions, the TCF network could be coupled with assessment of fMRI measures of whole brain functional connectivity to better understand how the tumor interacts with and may alter network connectivity in normal brain tissue within tumor and contralateral hemispheres.

Current mpMRI quantitative metrics include the Apparent Diffusion Coefficient (ADC) map values and Fractional Anisotropy (FA) derived from DWI/DTI imaging for brain, breast, and prostate imaging. The ADC metric characterizes the cellularity and flow of water in benign or malignant tumors, whereas FA depicts the amount of anisotropy (directionally) within different tissue types [53,54,55,56,57]. Reports have shown that ADC values for malignant tumors are typically significantly lower compared to benign lesions [37,58,59,60,61]. For example, HGG have significantly lower ADC map values than LGG, and both malignant breast and prostate lesions have lower ADC map values compared to benign lesions. Correspondingly, the tumor connectomics reveal a similar structure with the degree centrality metrics from the TCF with ADC map values across brain, breast, and prostate lesions. For example, benign breast lesions demonstrated significantly higher connectivity (degree centrality) than malignant breast lesions (lower degree centrality), which may be due to a more homogeneous nature of benign lesions, compared to the more heterogenous malignant lesions. Benign lesions appear to cluster together more as shown by the significantly smaller number of subgraphs or clusters present in the benign tumor connectomes, compared to malignant breast connectomes. 

Similarly, HGG tumors exhibited a lower degree centrality and clustering coefficient than LGG tumors. These differences could be attributed to more heterogeneity in contrast enhancement as well as irregular centrally necrotic areas in HGG brain tumors. As with malignant breast lesions, the large central necrotic regions of HGG likely demonstrate higher average path lengths due to the very low (actually, zero) cellular density in these necrotic regions that reduce overall connectivity among more spatially distant peripheral hypercellular regions. This is in contrast to LGG that rarely demonstrate internal necrosis and display relatively more homogeneous appearance on both T_1_-weighted and T_2_/FLAIR images. Thus, these graph metrics allow for the interrogation of the topology and dynamics of the TCF in the tumor microenvironment. As with breast and G7 prostate lesions, HGG demonstrated higher average path lengths within the lesion and surrounding tissue that are likely due to the extremely low cellular density within nonenhancing central necrotic regions that may reduce overall connectivity among more spatially distant peripheral hypercellular enhancing regions. Longitudinal assessments of such regional differences in tumor microenvironment may allow for more effective targeted therapeutic interventions and may be worth exploring in future work. The prostate tumor connectomics displayed similar characteristics as the breast tumor connectomics, where in the normal tissue regions displayed a higher betweenness compared to the tumor regions and the average path lengths were similar across the different malignant lesions. Prostate tumors with Gleason 6 demonstrated a higher connectivity than prostate tumors Gleason 7, characterized by increased degree centrality, eigenvector centrality, and clustering coefficient.

These preliminary results indicate a generalizability of the tumor connectomics to different organs and demonstrate the capability of this approach to assess more intricate organization of the tumor microenvironment. In summary, all three cancer types, in general, demonstrated similar characteristics, where in, more aggressive cancers demonstrated a lower connectivity than less aggressive cancers, demonstrating the stability and reliability of connectomics across different cancer types.

Limitations of using the TCF for evaluation and visualization of tumor connectomics may lie in computational complexity. The computationally expensive nature of the analysis in terms of space complexity of O (N^2^) and time complexity of O (N^3^). However, these computational limitations could be overcome by using advanced approximation methods and streaming algorithms to make TCF more computationally efficient and are under investigation [62,63,64,65]. This study was retrospective in nature with no longitudinal analysis of tumor growth or reduction after intervention to gauge treatment response and associated changes in the TCF in a prospective manner. However, there is ongoing work to investigate the changes in the TCF in tumors after different types of treatment. Moreover, larger prospective studies are needed to compare the TCF with other methods for classification of tumors into benign or malignant categories, including incorporation of molecular markers in tumor assessment, as well as for evaluation of treatment response.

## 5. Conclusions

The TCF provide novel visualization and classification metrics for different tumor types while providing characterization of the complex networks between the peritumoral and intratumoral regions, thereby permitting a better understanding of the global and regional connections within the lesion and surrounding tissue.

## Figures and Tables

**Table 1:** Summary of Demographic, Pathological and Clinical Data.

Malignant Characteristics	IDC	DCIS + IDC	IDC + ILC	ILC	Sarcomatoid	
	N = 20 (33%)	N = 17 (28%)	N = 14 (23%)	N = 7 (13%)	N = 1 (3%)	
Age, years ^a^	53 ± 11	52 ± 10	56 ± 12	58 ± 6	68	
**Phenotype**						
Luminal A ^b^	9	4	8	4	0	
Luminal B ^b^	7	7	5	4	0	
HER2+ ^b^	1	0	0	0	0	
Triple Negative ^b^	3	6	1	0	1	
Benign Characteristics	Benign Breast Tissue	Stable Imaging	Fibroadenoma	ADH	ADH with Lobular Features	Papilloma
	N = 12 (30%)	N = 9 (23%)	N = 8 (20%)	N = 6 (13%)	N = 4 (10%)	N = 2 (5%)
Age, years ^a^	49 ± 9	52 ± 6	44 ± 14	55 ± 6	49 ± 9	63 ± 10

^a^ Data are presented as mean ± (standard deviation); ^b^ Data are presented as number of cases. DCIS = Ductal carcinoma in situ; ILC = Invasive lobular carcinoma; LCIS = Lobular carcinoma in situ; IDC = Invasive ductal carcinoma, ADH = Atypical Ductal Hyperplasia, HER2+ = human epidermal growth factor receptor 2.

**Table 2:** Summary of the centrality metrics, average path length, and the number of clusters for the tumor connectomes modeled for benign and malignant breast lesions. Results are shown with mean with standard deviation of the mean. MCC = Matthew Correlation Coefficient, AUC-PR = Precision Recall with Bootstrap Confidence Interval (CI).

	Degree Centrality	Betweenness Centrality	Eigenvector Centrality	Average Path Length	Number of Connected Components	IsoSVM
**Benign**	0.14 ± 0.08	0.02 ± 0.02	0.01 ± 0.01	2.73 ± 0.93	6.10 ± 8.42	−1.55 ± 1.66
**Malignant**	0.07 ± 0.06	0.01 ± 0.01	0.004 ± 0.01	3.28 ± 0.91	10.76 ± 11.35	0.24 ± 1.22
***p* value**	0.000002	0.002	0.00001	0.20	0.02	0.000001
**MCC**	0.54	0.29	0.48	0.35	0.43	0.63
**AUC-ROC**	0.79 (95% CI = 0.70–0.89)	0.68 (95% CI:0.58–0.78)	0.76 (95% CI = 0.67–0.85)	0.64 (95% CI = 0.37–0.90)	0.69 (95% CI = 0.58–0.80)	0.86 (95% C = 0.76–0.92)
**AUC-PR**	0.83 (95% CI = 0.72– 0.90)	0.73 (95% CI = 0.64–0.82)	0.78 (95% CI = 0.70–0.86)	0.46 (95% CI = 0.22–0.83)	0.73 (95% CI = 0.62–0.82)	0.90 (95% CI = 0.79–0.95)

**Table 3:** Summary of the centrality metrics, clustering coefficient, and average path length for the tumor connectomes modeled for WHO Grade 2 and 4 brain tumors. Results are shown with mean with standard deviation. MCC = Matthew Correlation Coefficient, AUC-PR = Precision Recall, Confidence Interval (CI).

	Degree Centrality	Betweenness Centrality	Eigenvector Centrality	Clustering Coefficient	Average Path Length	IsoSVM
**Grade 2**	0.74 ± 0.15	0.0001 ± 0.0001	0.0005 ± 0.0003	0.90 ± 0.04	1.26 ± 0.16	−1.44 ± 1.24
**Grade 4**	0.56 ± 0.17	0.0004 ± 0.0004	0.0005 ± 0.0006	0.84 ± 0.06	1.59 ± 0.30	0.04 ± 1.18
***p* value**	0.02	0.07	0.68	0.0008	0.002	0.01
**MCC**	0.65	0.50	0.45	0.31	0.60	0.55
**AUC-ROC**	0.78 (95% CI = 0.59–0.97)	0.66 (95% CI = 0.43–0.88)	0.55 (95% CI = 0.31–0.80)	0.80 (95% CI = 0.63–0.98)	0.85 (95% CI = 0.69–1.00)	0.85 (95% CI = 0.65–0.96)
**AUC-PR**	0.87 (95% CI = 0.70–0.96)	0.81 (95% CI = 0.66–0.92)	0.74 (95% CI = 0.56–0.87)	0.90 (95% CI = 0.77–0.97)	0.93 (95% CI = 0.82–0.98)	0.92 (95% CI = 0.64–0.99)

**Table 4:** Summary of the centrality metrics, average path length, clustering coefficient, and apparent diffusion coefficient (ADC) map values corresponding to normal and lesion tissue regions within the prostate. Results are shown with mean with standard deviation.

Tissue	Degree Centrality	Betweenness Centrality	Eigenvector Centrality	Clustering Coefficient	Average Path Length	ADC Value (×10^−3^ mm^2^/s)
**Normal**	0.83 ± 0.11	0.001 ± 0.001	0.01 ± 0.004	0.93 ± 0.04	1.18 ± 0.12	1.44 ± 0.21
**Lesion**	0.90 ± 0.10	0.0003 ± 0.0003	0.01 ± 0.01	0.96 ± 0.04	1.11 ± 0.14	0.83 ± 0.15
***p* value**	0.10	0.005	0.6	0.01	0.10	>0.0002

**Table 5:** Summary of the centrality metrics, average path length, clustering coefficient and apparent diffusion coefficient (ADC) values corresponding to comparison of normal and lesion regions for G6 versus G7 prostate cancer. Results are shown with mean with standard deviation of the mean. MCC = Matthew Correlation Coefficient, PR = Precision Recall.

Gleason Score	Degree Centrality	Betweenness Centrality	Eigenvector Centrality	Clustering Coefficient	Average Path Length	ADC Value (× 10^−3^ mm^2^/s)
**6**	0.98 ± 0.02	0.0001 ± 0.0001	0.02 ± 0.01	0.99 ± 0.01	1.00 ± 0.03	0.89 ± 0.67
**7**	0.83 ± 0.09	0.0005 ± 0.0004	0.002 ± 0.001	0.93 ± 0.03	1.21 ± 0.13	0.81 ± 0.17
***p* value**	0.001	0.04	0.03	0.0002	0.002	0.28
**MCC**	0.87	0.73	0.71	0.87	0.87	0.55
**AUC-ROC**	0.98 (CI = 0.92–1.00)	0.88 (CI = 0.70–1.00)	0.92 (CI = 0.77–1.00)	0.98 (CI = 0.92–1.00)	0.98 (CI =0.92–1.00)	0.69 (CI = 0.37–1.00)
**AUC-PR**	0.99 (Bootstrap) CI = 0.87–1.00)	0.91 (Bootstrap) CI = 0.70–1.00)	0.94 (Bootstrap) CI = 0.73–1.00)	0.99 (Bootstrap) CI = 0.87–1.00)	0.99 (Bootstrap) CI = 0.88–1.00)	0.83 (Bootstrap) CI 0.61–0.97)

**Table 6:** Summary centrality metrics, clustering coefficient, and average path length for the tumor connectomes modeled from the validation I-SPY breast dataset. Results are shown with mean with standard deviation. CR = Clinical Response, MCC = Matthew Correlation Coefficient, AUC-PR = Precision Recall with Bootstrap Confidence Interval (CI).

Response	Degree Centrality	Betweenness Centrality	Eigenvector Centrality	Average Path Length	Number of Connected Components	IsoSVM
**CR = 1,2**	0.01 ± 0.25	0.53 ± 0.51	0.49 ± 0.38	0.01 ± 0.16	−0.03 ± 0.13	1.50 ± 1.96
**CR = 3,4**	0.07 ± 0.22	−0.05 ± 0.50	0.05 ± 0.56	−0.04 ± 0.13	0.11 ± 0.33	−0.78 ± 2.53
***p* value**	0.000002	0.01	0.05	0.35	0.26	0.03
**MCC**	0.29	0.67	0.50	0.34	0.29	0.70
**AUC-ROC**	0.62 (95% CI = 0.44–0.79)	0.84 (95% CI = 0.67–0.94)	0.76 (95% CI = 0.58–0.89)	0.65 (95% CI = 0.46–0.84)	0.57 (95% CI = 0.39–0.74)	0.87 (95% C = 0.71–0.96)
**AUC-PR**	0.78 (95% CI = 0.58–0.90)	0.92 (95% CI = 0.73–0.98)	0.85 (95% CI = 0.66–0.95)	0.85 (95% CI = 0.65– 0.95)	0.79 (95% CI = 0.59–0.91)	0.92 (95% CI = 0.73–0.98)

**Table 7:** Summary for the validation BRATS dataset for low grade gliomas (LGG) and high grade gliomas (HGG) with the centrality metrics, clustering coefficient, and average path length for the tumor connectomes modeled. Results are shown with mean with standard deviation. MCC = Matthew Correlation Coefficient, AUC-PR = Precision Recall with Bootstrap Confidence Interval (CI).

Glioblastoma	Degree Centrality	Betweenness Centrality	Eigenvector Centrality	Node Strength	Average Path Length	IsoSVM
**Low Grade**	0.61 ± 0.15	0.00035 ± 0.00030	0.00021 ± 0.0003	0.10 ± 0.03	1.55 ± 0.31	−0.56 ± 1.04
**High-Grade**	0.47 ± 0.17	0.00044 ± 0.00033	0.00042 ± 0.0004	0.13 ± 0.04	1.88 ± 0.41	0.53 ± 1.06
***p* value**	0.000001	0.0025	0.0001	0.0001	0.0001	0.0001
**Sensitivity (%)**	67	77	77	68	71	72
**Specificity (%)**	75	43	63	72	71	75
**MCC**	0.41	0.22	0.19	0.40	0.41	0.46
**AUC-ROC**	0.75 (CI = 0.70–0.79)	0.61 (CI = 0.56–0.65)	0.57 (CI = 0.53–0.62)	0.74 (CI = 0.69–0.77)	0.74 (CI = 0.70–0.78)	0.77 (CI = 0.73–0.81)
**AUC-PR**	0.82 (CI = 0.77–0.86)	0.67 (CI = 0.61–0.72)	0.61 (CI = 0.55–0.67)	0.80 (CI = 0.75–0.85)	0.80 (CI = 0.74–0.84)	0.82 (CI = 0.77–0.86)

## Data Availability

The test brain and breast image data are from studies conducted by the investigators and availability is determined by the institution. The prostate data set is available upon request from the investigators. The validation data sets can be accessed at The Cancer Imaging Archive (TCIA)—https://www.cancerimagingarchive.net (accessed on 13 November 2021).
